# Effect of frenotomy on breastfeeding variables in infants with ankyloglossia (tongue-tie): a prospective before and after cohort study

**DOI:** 10.1186/s12884-017-1561-8

**Published:** 2017-11-13

**Authors:** Kathryn Muldoon, Louise Gallagher, Denise McGuinness, Valerie Smith

**Affiliations:** School of Nursing & Midwifery, Trinity College Dublin, 24 D’Olier Street, Dublin, D02 T283 Ireland

**Keywords:** Breastfeeding, Ankyloglossia, Tongue-tie, Frenotomy

## Abstract

**Background:**

Controversy exists regarding ankyloglossia (tongue-tie) and its clinical impact on breastfeeding, including the benefits, or otherwise, of tongue-tie release (frenotomy). As exclusive breastfeeding rates in Ireland are already considerably low (46% on discharge home from the maternity unit following birth in 2014), it is imperative to protect and support breastfeeding, including identifying the associated effects that frenotomy might have on breastfeeding variables.

**Objective:**

To determine the associated effects of frenotomy on breastfeeding variables in infants with ankyloglossia.

**Methods:**

A prospective before and after cohort study was conducted. Following ethical approval, two self-reported questionnaires were administered to women whose infants were undergoing frenotomy at seven healthcare clinics in the Republic of Ireland. Data on breastfeeding variables prior to the frenotomy procedure and at 1-month post-frenotomy were collected and compared. Descriptive statistics (frequencies and proportions) were used to analyse, separately, the pre- and post-frenotomy data. Inferential statistics (z-test scores for differences between proportions (alpha <0.05) and mean differences (MD) with 95% confidence intervals (CI)) were used for pre- and post-frenotomy comparative analyses.

**Results:**

Ninety-eight women returned the baseline questionnaire, and, of these, 89 returned the follow-up questionnaire. The most common reason for seeking a frenotomy was difficulty with latch (38%). Private lactation consultants were the *main* person recommending a frenotomy (31%). Rates of exclusive breastfeeding remained similar pre- and post-frenotomy (58% versus 58%), although rates of formula feeding increased two-fold at follow-up. Infants’ ability to extend their tongues to the lower lip after frenotomy was significantly increased (*p* < 0.0001)*.* Almost all participants (91%) reported an *overall* improvement in breastfeeding post-frenotomy. Pain on breastfeeding was significantly reduced post-frenotomy (MD 2.90, 95% CI 3.75 to 2.05) and overall LATCH scale scores were significantly increased (MD -0.50, 95% CI -0.67 to −0.33).

**Conclusions:**

This study supports the hypothesis that frenotomy has a positive effect on breastfeeding variables in infants with ankyloglossia. These findings, however, are based on a relatively small number of participants from one country only where breastfeeding rates are low. Further, larger studies are required to substantiate these findings.

**Electronic supplementary material:**

The online version of this article (doi: 10.1186/s12884-017-1561-8) contains supplementary material, which is available to authorized users.

## Background

Ankyloglossia (tongue-tie) is a congenital anomaly that occurs when infants are born with an abnormally short lingual frenulum which results in restricted tongue movement [1]. This restriction may include limited forward protrusion of the tongue or reduced lateral mobility of the tongue [2]. The prevalence of tongue-tie varies across studies and settings; for example, a methodological review cites rates of between 4.2 and 10.7% based on five included studies [3]. In a more recent study (2014) involving school children in India, a significantly higher rate of 16.4% was reported [4]. While reasons for variations are not always clear they are likely explained by differences in age groups across studies (i.e. from newborns [5] to young adults of up to 17 years of age [4]) and by differences in how the tongue-tie was diagnosed (i.e. by clinician subjective assessment or by using objective measurement instruments, such as KOTLOW’s method of grading [4] or Hazelbaker’s assessment tool [6]). Poor latching of the infant to the breast, weight issues due to poor milk transfer, frequent feeding at the breast and persistent sore and cracked nipples have been reported by women as breastfeeding difficulties associated with tongue-tie [7, 8]. As breastfeeding benefits both a mother and her infant, it is essential that any condition which may potentially affect successful breastfeeding in women who chose to breastfeed be considered a public health issue that should be addressed [9].

Frenotomy is a simple surgical procedure whereby the frenulum is divided either in the midline or at the underside of the tongue using a sharp blunt ended scissors [8–10]. During the procedure the infant is swaddled in a blanket, generally no anaesthesia is administered and there is reportedly little bleeding or few complications following the procedure [9–11].

Frenotomy was widely practised by midwives and physicians up to the twentieth century until bottle feeding became the norm in Western culture [8]. In more recent times, however, parents describe conflicting advice regarding the management of tongue-tie. In an Australian study, for example, postpartum women reportedly encountered health professionals who had limited knowledge of tongue-tie or how it impacted on breastfeeding experiences [12]. Women in this study described a challenging journey in establishing breastfeeding which was in contrast to the natural experience they had anticipated. This experience is mirrored in Ireland where women also describe challenges with breastfeeding with many also experiencing a lack of support and expert advice from health professionals [13]. This, in turn, presents challenges for families with decision-making on whether to opt, or not, for a frenotomy. For women in the Republic of Ireland (ROI), where frenotomy is mainly performed privately by independent clinicians remote from the maternity hospital setting [14], the cost of the procedure is one of the main challenges that women may encounter.

There is currently conflicting evidence in relation to the benefits, or otherwise, of tongue-tie release. The Canadian Paediatric Society and the Japanese Paediatric Society state that tongue-tie does not present breastfeeding challenges for all infants and do not recommend routine frenotomy [1, 15]. Contrastingly, others suggest that tongue-tie can affect breastfeeding and that a frenotomy procedure will increase infants’ ability to breastfeed successfully, immediately, and for longer durations [16–18]. Guidelines from the National Institute of Health and Care Excellence (NICE), while acknowledging that there is limited evidence to suggest that frenotomy can improve breastfeeding, state also that the available evidence is *“adequate to support the use of the procedure provided that normal arrangements are in place for consent, audit and clinical governance”* and suggest that breastfeeding challenges can be resolved following the procedure [10]. These guidelines, however, have been criticised for being dated [19].

With low exclusive breastfeeding rates in Ireland (46% on discharge home from the maternity unit following birth (1–4 days postpartum) in 2014) [20], and limited evidence on the possible effects of frenotomy on breastfeeding variables from the perspectives of women who are breastfeeding, further research that has the potential to inform, protect and support breastfeeding, is required.

## Aim

To determine associated effects of frenotomy on breastfeeding variables in infants with ankyloglossia.

### Objectives

1. To determine breastfeeding variables at baseline prior to frenotomy in infants with tongue-tie;
2. To determine if frenotomy has an impact on breastfeeding variables in infants with tongue-tie, and explore whether such impacts are positive or negative;
3. To explore and determine factors that influence breastfeeding women’s decision to choose frenotomy for their infants.

## Methods

### Study design

A prospective before and after cohort study was conducted. Breastfeeding variables were determined at baseline prior to the frenotomy procedure and at 1-month post-frenotomy, and compared.

### Participants

Eligible participants were postpartum women, 18 years of age or older, who were breastfeeding their infants and attending a healthcare clinic for the purpose of a planned frenotomy. Due to a lack of available national data on the numbers of infants undergoing frenotomy we were unable to calculate a sample size for the study. Alternatively, we pragmatically aimed to recruit as many participants as possible over the 5 months study period (March to July 2016).

### Setting and data collection

Two self-reported questionnaires were used to collect the data. A baseline (pre-frenotomy) questionnaire (Additional file 1) was administered to potential participants via seven healthcare clinics/GP practices in the ROI where frenotomy is performed. These clinics are external to the maternity hospitals as community/primary care services with minimal data linkage between postpartum and follow-up infant care. For this reason, we are unable to ascertain precise overall tongue-tie incidence rates, however, we are aware that frenotomy procedures varied across settings and have described these in Table 1. Women who were attending for frenotomy were provided with the study information leaflet, consent form and the baseline questionnaire. For those who wished to take part a request was made to return the questionnaire and their signed consent form, by post, within 1 week of the frenotomy procedure. A stamped addressed envelope was provided for this purpose. A follow-up questionnaire (Additional file 2) was posted one-month later to those women who returned the baseline questionnaire with a request for return within 1 week, or earlier, from receiving the questionnaire. Baseline and follow-up questionnaires sought similar information to allow for comparisons on breastfeeding variables pre- and post- the frenotomy procedure.

**Table 1:** Participant Demographics

Variable	Results
Frenotomy practitioners	
Paediatrician/Paediatric Surgeon	2
General Practitioner	3
Specialist Oral/ENT Surgeon	1
Frenotomy procedure	Varied
Analgesia	No analgesia or 5% lignocaine oral gel, topical local anaesthetic gel (in infants >3 months), 24% sucrose solution in younger babies, or oral sucrose at >8 weeks prior to the procedure; paracetamol and ibuprofen as required afterwards
Scissors used	Metzenbaum scissors, strabismus scissors or single-use Iris scissors
First baby (n, %)	
Yes	37 (38%)
No	61 (62%)
Family history of tongue-tie (n, %)	
Yes	40 (41%)
No	58 (59%)
Age of infants at time of frenotomy	
Mean (SD)	7 weeks, 3 days (6 weeks, 2 days)
Range	1 week, 0 days to 25 weeks, 5 days
Age of infants at time of follow-up	
Mean (SD)	11 weeks, 6 days (6 weeks, 1 days)
Range	4 weeks, 0 days to 32 weeks, 6 days

### Instrument genesis

The questionnaires were initially generated from a review of the literature and from clinical expertise within the research team (lactation consultant). Information sought on the questionnaires included; participant demographics (parity, history of tongue-tie, age of infant at frenotomy, etc.), method of feeding pre-frenotomy and at follow-up, main reasons for choosing a frenotomy, main person recommending frenotomy, infant tongue extension on latch, challenges/concerns regarding tongue-tie, pain on breastfeeding and breastfeeding-specific variables such as latch, nipple shape and infant satiation after feeding. Pain on breastfeeding was measured using a standard 0–10 visual analogue scale, where 0 was *‘no pain’* and 10 was *‘extremely severe pain’*. Breastfeeding-specific variables were measured using a modified version of the LATCH scale [21], which was used with permission and validated (content relevance) for use in an Irish population prior to distribution. The items in the scale are latch, audible swallowing, type of nipple (e.g. inverted, flat), nipple shape, breast (engorged, firm), nipple (cracked, bleeding, intact), urine characteristics and infant satiation. The original LATCH scale contained the first 5 items only [21]. The latter two items were later added to enhance the use of the LATCH tool in clinical practice (C. Davis, personal communication) following an assessment by Riordan and Koehn [22] that questioned the reliability of the LATCH tool, by demonstrating interrater reliability scores of between 0.11 and 0.46, for assessing breastfeeding [22]. Within each item of the scale there are three response options scored 1–3. Higher individual-item and overall scale scores have been associated with longer breastfeeding durations [23]. All remaining items in the surveys underwent face and content validity testing by a panel of experts (2 lactation consultants, 3 midwife researchers, 1 clinician who performs frenotomies and 10 women who were breastfeeding) and were amended accordingly prior to distribution. Testing revealed that the questionnaires took less than 10 min for the mother to complete.

### Data analysis

Descriptive statistics (frequencies, means and standard deviations) were used to describe, separately, the results of the baseline questionnaire and the 1-month follow-up questionnaire. Inferential statistics (z-test scores for differences in proportions and mean differences with 95% confidence intervals) were used to determine differences between pre- and post-frenotomy with an alpha of <0.05 indicating statistical significance.

### Ethical approval

Ethical approval to conduct the study was granted by the Research Ethics Committee of the School of Nursing & Midwifery, Trinity College Dublin. Written consent to participate in the study was returned by participants with their baseline questionnaire. All information was stored in accordance with the Data Protection (Amended) Act 2003 [24].

## Results

### Participant demographics

Two-hundred and eighty-one baseline questionnaires were distributed to women whose babies were undergoing a frenotomy procedure across the seven healthcare clinics. Ninety-eight women (35%) completed and returned the baseline questionnaire and, of these, 89 (91%) completed the follow-up questionnaire. Table 1 provides the participant’s demographic details and information on the frenotomy practitioners and frenotomy procedures.

### Reasons for seeking frenotomy

The most common reported reason for seeking a frenotomy was difficulty latching baby to the breast (38%, *n* = 37) followed by nipple pain (20%, *n* = 19). Other reasons included baby unsettled post-feed (10%, *n* = 10), concern over later speech (6%, *n* = 6), difficulty maintaining latch (5%, *n* = 5), mastitis (3%, n = 3), breast feeling full post-feed (1%, n = 1), concern over weight gain (3%, n = 3), previous infant had tongue-tie and feeding improved after frenotomy (3%, n = 3) and concern over milk supply (2%, *n* = 2).

### Main person recommending frenotomy

**Fig. 1:**
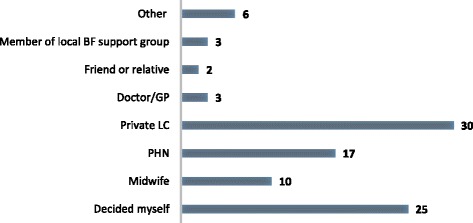
Main person recommending a frenotomy

Private lactation consultants (LCs) that is International Board Certified Lactation Consultants (IBCLCs) (31%; *n* = 30) followed closely by women deciding themselves (26%; *n* = 25) were the *main* persons recommending a frenotomy procedure (Fig. 1).

### Feeding method pre- and post-frenotomy

Rates of exclusive breastfeeding remained similar between pre-frenotomy and at follow-up, although rates of formula feeding increased two-fold at follow-up (Table 2).

**Table 2:** Infant feeding pre- and post-frenotomy

Method of Feeding	Pre- N/98 (%)	Post- N/89 (%)	*z*-score	*p*-value*
Exclusive breastfeeding	57 (58%)	52 (58%)	−0.04	0.97
Expressing breast milk (feeding using a bottle)	2 (2%)	2 (2%)	−0.09	0.92
Combination breastfeeding and expressed breast milk	17 (17%)	9 (10%)	1.43	0.15
Combination breastfeeding and formula	12 (12%)	14 (16%)	−0.68	0.49
Formula feeding	6 (6%)	12 (13%)	−1.7	0.89

*significant at p = <0.05

For women who indicated that they were no longer breastfeeding at follow-up, Table 3 provides examples of the primary, secondary and tertiary reasons why they decided to stop breastfeeding.

**Table 3:** Main reasons women decided to stop breastfeeding after frenotomy

Primary reasons 11 responses	Secondary reasons 9 response	Tertiary reasons 8 response
Couldn’t latch correctly (n = 2) Jaundice levels dangerous (*n* = 1) Too painful/nipple pain (*n* = 1) No improvement post-frenotomy (*n* = 1) Low/insufficient supply (*n* = 3) Nipple painful/bleeding (n = 1) Tongue-tie reoccurred (*n* = 2)	Feeding every 2 h (n = 1) Feeding too painful (n = 2) Mastitis (n = 1) Nipple damage (n = 1) Too stressful for mum & baby (n = 1) Unable to get off shield (n = 1) Weaning in progress (n = 1) Weight loss (n = 1)	Insufficient supply (n = 1) Baby never seems satisfied (n = 1) Baby very demanding (n = 1) Poor latch preventing bonding (n = 1) Returned to work (n = 1) Too tired (n = 1) Too costly to get LC help again (n = 1)

### Type of tongue-tie

Women were asked to identify the type of tongue-tie their infant had by indicating the picture, from a set of four pictures of types of tongue-tie, which were presented on the baseline questionnaire. These four pictures were based on Coryllos and colleagues classification of tongue-tie [25, 26] and used with permission. Eleven participants (11%) reported a Type 1 tongue-tie (classical tongue-tie with a heart shaped tongue), 45 (46%) reported Type 2 (restricted elevation and extension of tongue), 15 (15%) reported Type 3 (bunched configuration, mid-tongue pulled down during extension) and 24 (24%) reported Type 4 (fibrous attachment, asymmetry of tongue movement, narrow palate) [25].

### Tongue extension

**Fig. 2:**
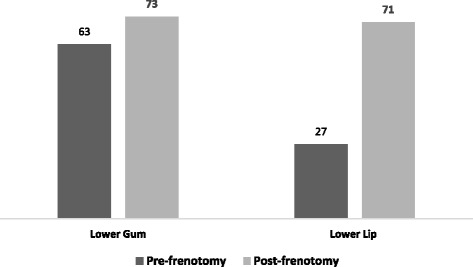
Ability to extend tongue

Infants’ ability to extend their tongues to both the lower gum and the lower lip was significantly increased post-frenotomy compared to pre-procedure (*p* = 0.006 and *p* < 0.0001, respectively) (Fig. 2).

### Improvement in breastfeeding after frenotomy

Almost all women (91%; *n* = 81/89) reported an *overall* improvement in breastfeeding after the frenotomy procedure. Forty-five percent (*n* = 40) experienced improvement immediately after the procedure and 29% (*n* = 26) experienced improvement within 2 weeks of the procedure. Four women (5%), only, reported no improvement in breastfeeding after the frenotomy.

### Specific concerns related to infants tongue-tie

**Fig. 3:**
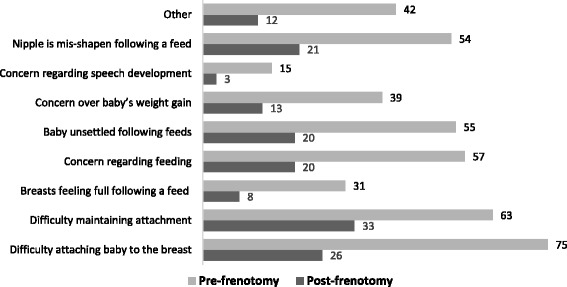
Concerns pre- and post-frenotomy

When women were asked to indicate if they were experiencing specific concerns related to their infant’s tongue-tie, these concerns were reported as all being significantly improved (*p* < 0.05 for all variables) after the frenotomy procedure (Fig. 3). Examples of *‘Other’* concerns include bleeding and blistered nipples (*n* = 8), clicking sound or coughing when feeding (*n* = 2), extreme pain/nipple pain when feeding (*n* = 14), milk running down baby’s face (*n* = 1) and needing to use nipple shields all of the time (*n* = 1).

### Sources of help with breastfeeding

**Fig. 4:**
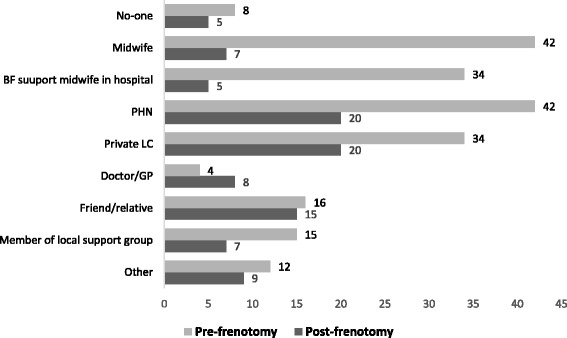
Sources of help for breastfeeding

Private lactation consultant (LC), public health nurse (PHN) and midwife were the persons most commonly helping women with breastfeeding challenges prior to frenotomy. Although private LC and PHN remained considerable sources of help for women post-frenotomy, women were more often helped by friends and relatives or by their doctor/GP after the frenotomy procedure compared to pre-procedure (Fig. 4).

### Pain on breastfeeding

Women were asked to rate their pain on breastfeeding using a visual analogue scale where 0 = no pain and 10 = extremely severe pain. Pain scores were significantly higher pre-frenotomy compared to post-frenotomy (pre-frenotomy mean 5.6 (SD 3.3) versus post-frenotomy mean 2.7 (SD 2.6); MD -2.90, 95% CI -3.75 to −2.05).

### Breastfeeding assessment using the LATCH scale

Ninety-two and 62 women completed the LATCH scale pre-frenotomy and at follow-up, respectively. Overall LATCH scale scores demonstrated a statistically significant improvement in breastfeeding variables post-frenotomy procedure (MD 0.50; 95% CI 0.33 to 0.67). Significant improvements were also found on all individual scale items other than type of nipple (Table 4).

**Table 4:** LATCH scale scores

	Pre-frenotomy (*n* = 92) Mean (SD)	Post-frenotomy (*n* = 62) Mean (SD)	Significance^a^ MD (95% CI)
Latch	2.1 (0.6)	2.7 (0.5)	0.6 (0.43 to 0.77)^a^
Audible swallowing	2.5 (0.6)	2.8 (0.5)	0.3 (0.12 to 0.47)^a^
Type of Nipple	2.9 (0.3)	2.9 (0.3)	N/A
Nipple Shape	1.7 (0.8)	2.5 (0.7)	0.8 (0.56 to 1.04)^a^
Breast	2.1 (0.8)	2.8 (0.4)	0.7 (0.51 to 0.89)^a^
Nipple	2.1 (0.8)	2.7 (0.6)	0.6 (0.38 to 0.82)^a^
Urine	2.8 (0.5)	3.0 (0.1)	0.2 (0.09 to 0.31)^a^
Satiation	2.0 (0.9)	2.8 (0.5)	0.8 (0.58 to 1.02)^a^
Overall LATCH Scale Scores	2.3 (0.7)	2.8 (0.4)	0.50 (0.33 to 0.67)^a^

^a^indicates significantly favouring post-frenotomy

## Discussion

This study provides important insights from the perspectives’ of women who are breastfeeding on the impact of frenotomy on breastfeeding variables, thus adding to the international body of evidence on this topic. Overall, the study’s findings demonstrate that frenotomy procedure positively affects breastfeeding variables. This compares favourably with evidence from previous studies [27, 28] and supports the current NICE guideline recommendation [10].

Overwhelmingly, almost all of the women in our study reported an overall improvement in breastfeeding and in specific challenges such as attachment and baby settling post-feed post-frenotomy. This has clinical importance for encouraging breastfeeding continuation as per international recommendations [29] and in supporting women who are experiencing difficulties to continue to breastfeed. The finding that private LCs were the main persons influencing women’s decision-making on frenotomy reflects the LC service in the ROI whereby only those women who can afford to pay for the service are able to avail of it. This creates disparity and inequality and suggests that midwives and other health care practitioners need to assume a greater role in discussing frenotomy with women in supporting them through the decision-making process and in assisting them with.

Significant improvements were found on five of the six individual LATCH scale items and the overall LATCH scale score was significantly higher post-frenotomy. This finding is consistent with previous studies using the original five item scale [2, 27, 30] although one study found no difference in overall LATCH scores at 8 weeks between infants who had a frenotomy at 5 days old and those who received standard breastfeeding support [11]. Most women in the comparison group, however, went on to seek a frenotomy due to pain during feeding [11], which is a feature common to other trials on frenotomy [28].

Critically, the results of our study demonstrate a significant reduction in self-reported pain scores post frenotomy. This is an important finding considering that painful breastfeeding and difficulty latching have been identified as significant predictors of early discontinuation of breastfeeding [31–33]. While rates of exclusive breastfeeding remained similar between pre-frenotomy and follow-up formula feeding increased two-fold at follow-up with many women who were combining breastfeeding with expressing breastmilk prior to the procedure switching to formula feeding post procedure. Combining breastfeeding with formula has been highlighted as a risk factor for discontinuation of breastfeeding [34] and while 91% of women in our study reported an overall improvement in breastfeeding, we also recognise that frenotomy alone will not resolve breastfeeding challenges for all mother infant dyads.

The majority of women in our study described experiencing difficulty with at least one, if not more than one, breastfeeding variable(s) that might have been directly associated with the tongue-tie. This is supported by the study data which indicates significant improvements in breastfeeding concerns/difficulties post-frenotomy. In particular, women experienced improvements with attaching the baby to the breast and maintaining attachment, both key aspects of successful breastfeeding [35].

### Strengths and limitations

There are a number of strengths and limitations associated with this study that need to be acknowledged. Firstly, due to a lack of national data we were unable to calculate a sample size for the study and recruitment and response was time-bound over 5 months. Acknowledging this, however, this study provides insight into the numbers of frenotomies being performed in the ROI which were higher than we had originally anticipated (i.e. at least 281 in seven primary care units/GP practices in 5 months). These numbers may provide a basis from which to estimate sample sizes for a larger prospective national study in the future. Maternal self-report strengthens the findings of this study, as women who are experiencing, first-hand, challenges with breastfeeding are best placed to notice any changes to these. Self-report and the nature of our survey does, however, involve a level of recall that is known to introduce bias. We attempted to minimise this, in as far as possible, by i) asking women to complete the baseline questionnaire as close to their infant’s frenotomy as possible and no longer than a week following the procedure, and ii) requesting that women consider breastfeeding in the 24–48 h prior to completing the follow-up questionnaire irrespective of the date they received it. Lastly, while our initial response rate of 35% was disappointing, although not unusual for postal questionnaires, participants’ follow-up response rates were excellent at 91%, highlighting, perhaps, that for many women the issue of tongue-tie, frenotomy and breastfeeding is of considerable importance to them.

### Implications for practice

Acknowledging this as a study from one country where exclusive breastfeeding rates are relatively low the findings have considerable relevance for practice internationally. In particular, as the study presents women’s self-reported experiences of changes in breastfeeding variables after frenotomy it provides further evidence, from the perspectives of women who are breastfeeding, on associated effect. The findings of this study may help other women who have infants with tongue-tie and reassure them that concerns and difficulties in feeding infants with tongue-tie are not uncommon. Furthermore, this study provides information for healthcare practitioners (midwives, PHNs, GPs, etc.,) who may be asked by women for information on frenotomy or asked to assist women in making decisions about the procedure. This study provides evidence to suggest that the majority of women experienced an overall improvement in breastfeeding after frenotomy and, in particular, pain scores were reduced. This information along with the study’s other findings may be relayed to women who seek advice on frenotomy to assist with any decisions that they might need/wish to make. Lastly, this study is one example of a prospective survey on the topic of tongue-tie, breastfeeding and frenotomy. The conduct of this study might provide an impetus for researchers in other countries to replicate the survey thus expanding the evidence base, internationally, on the associated effects of frenotomy on breastfeeding variables.

## Conclusions

This study supports the hypothesis that frenotomy positively affects breastfeeding variables in infants with tongue-tie. Further research, involving larger sample sizes, however, is required to substantiate these findings. Further qualitative studies that explore the effect of frenotomy on breastfeeding in infants who have tongue-tie, from the perspectives’ and experiences of women who were breastfeeding infants with tongue-tie, are also required.

## Additional files

Additional file 1: Final baseline questionnaire. Questionnaire used to collect information on pre-frenotomy variables. (PDF 264 kb)

Additional file 2: Final follow-up questionnaire. Questionnaire used to collect information on post-frenotomy variables. (PDF 239 kb)

## Abbreviations

GP: General Practitioner
IBCLC: International Board Certified Lactation Consultant
LC: Lactation consultant
PHN: Public health nurse
ROI: Republic of Ireland

## References

[1] Rowan-Legg A (2015). Ankyloglossia and breastfeeding: position statement. Can Paediatr Soc Community Paediatr Committee.

[2] Geddes D, Langton D, Gollow I, Jacobs L, Hartmann P, Simmer K (2008). Frenulotomy for breastfeeding infants with ankyloglossia: effect on milk removal and sucking mechanism as imaged by ultrasound. Pediatrics.

[3] Segal LM, Stephenson R, Dawes M, Feldman P (2007). Prevalence, diagnosis, and treatment of ankyloglossia: Methodologic review. Can Fam Physician.

[4] Bai PM, Vaz AC (2014). Ankyloglossia among children of regular and special schools in karnataka, India: a prevalence study. J Clin Diagn Res.

[5] González JD, Costa RM, Riaño GI, González MMT, Rodríguez PMC, Lobete PC (2014). Prevalence of ankyloglossia in newborns in Asturias (Spain). An Pediatr (Barc).

[6] Ricke LA, Baker NJ, Madlon-Kay DJ, Defore DA (2005). Newborn tongue tie: prevalence and effect on breast-feeding. J Am Board Fam Pract.

[7] Health Service Executive (HSE). Management of tongue tie in early infancy. National Clinical Programme for Paediatrics and Neonatology Dublin. http://www.hse.ie/eng/about/Who/clinical/natclinprog/paediatricsandneonatology/resources/TongueTieinEarlyInfancy.pdf. Accessed 23 Nov 2016.

[8] Watson Genna C (2008). Supporting sucking skills in breastfeeding infants.

[9] Griffiths M (2004). Do tongue ties affect breastfeeding?. J Hum Lact.

[10] National Institute for Health and Care Excellence (NICE) (2005). Division of ankyloglossia (tongue-tie) for breastfeeding.

[11] Emond A, Ingram J, Johson D, Blair P, Whitelaw A, Copeland M, Sutcliffe A (2014). Randomised controlled trial of early frenotomy in breastfed infants with mild-moderate tongue-tie. Arch Dis Child Neonatal Ed.

[12] Edmunds J, Fulbrook P, Miles S (2013). Understanding the experiences of mothers who are breastfeeding an infant with tongue-tie. J Hum Lact.

[13] Glynn R, Colreavy M, Rowley H, Gendy S (2012). Division of tongue tie: review of practice through a tertiary paediatric otorhinolaryngology service. Int J Pediatr Otorhinolaryngol.

[14] Association for Improvement in the Maternity Services (AIMS) Ireland (2014). What matters to you survey 2014.

[15] Committee of Ethics, Japan Pediatric Society (2001). Survey on operative treatment in ankyloglossia and its results. J Japan Pediat Soc.

[16] Steehler M, Steehler M, Harley E (2012). A retrospective review of frenotomy in neonates and infants with feeding difficulties. Int J Pediatr Otorhinolaryngol.

[17] Dollberg S, Marom R, Botzer E (2014). Lingual frenotomy for breastfeeding difficulties: a prospective follow-up study. Breastfeed Med.

[18] Hogan M, Westcott C, Griffiths M (2005). Randomized, controlled trial of division of tongue-tie in infants with feeding problems. J Paediatr Child Health.

[19] Burrows S, Lanlehin R (2015). Is frenotomy effective in improving breastfeeding in newborn babies with tongue-tie? A literature review. British J Midwifery.

[20] Healthcare Pricing Office (2016). Perinatal statistics report 2014.

[21] Jenson D, Wallace S, Kelsay P (1994). LATCH: a breastfeeding charting system and documentation tool. JOGNN.

[22] Riordan JM, Koehn M (1997). Reliability and validity testing of three breastfeeding assessment tools. JOGNN.

[23] Riordan J, Bibb D, Miller M, Rawlins T (2001). Predicting breastfeeding duration using the LATCH breastfeeding assessment tool. J Hum Lact.

[24] Government of Ireland. Data Protection (Amended) Act 2003. http://www.irishstatutebook.ie/eli/2003/act/6/enacted/en/pdf. Accessed 12 Dec 2016.

[25] Watson G.C. Coryllos E.V. http://www.brianpalmerdds.com/pdf/cwatson_tongue_presentation.pdf. Accessed 10 Jan 2017.

[26] Coryllos E, Watson GC, Salloum A. Breastfeeding: best for baby and mother, congenital tongue-tie and its impact on breastfeeding. Newsl Am Acad Pediatr. 2004:1–2.

[27] Berry J, Griffiths M, Westcott CA (2012). Double-blind, randomized, controlled trial of tongue-tie division and its immediate effect on breastfeeding. Breastfeed Med.

[28] Buryk M, Bloom D, Shope T (2011). Efficacy of neonatal release of ankyloglossia: a randomized trial. Pediatrics.

[29] World Health Organisation (WHO) (2001). Global strategy for infant and young child feeding: the optimal duration of exclusive breastfeeding.

[30] Srinivasan A, Dobrich C, Mitnick H, Feldman P (2006). Ankyloglossia in breastfeeding infants: the effect of frenotomy on maternal nipple pain and latch. Breastfeed Med.

[31] Begley C, Gallagher L, Carroll M, Clarke M, Millar S. The National Infant Feeding Survey: Trinity College Dublin; 2008. https://nursing-midwifery.tcd.ie/assets/publications/pdf/report-of-the-national-infant-feeding-survey.pdf. Accessed 10 Jan 2017.

[32] McAndrew F, Thompson J, Fellows L, Large A, Speed M, Renfrew M (2012). Infant feeding survey 2010.

[33] Dennis C, Jackson K, Watson J. Interventions for treating painful nipples among breastfeeding women. Cochrane Database Syst Rev. 2014;(12):CD007366. doi:10.1002/14651858.CD007366.pub2. 10.1002/14651858.CD007366.pub2 PMC10885851 25506813

[34] Chantry C, Dewey K, Peerson J, Wagner E, Nommsen-Rivers L (2014). In-hospital formula use increases early breastfeeding cessation among first-time mothers intending to exclusively breastfeed. J Pediatr.

[35] Amir LH, Dennerstein L, Garland SM, Fisher J, Farish SJ (1996). Psychological aspects of nipple pain in lactating women. J Psychosom Obstet Gynaecol.

