# Supplementary material for: Effect of frenotomy on breastfeeding variables in infants with ankyloglossia (tongue-tie): a prospective before and after cohort study
Source: BMC Pregnancy Childbirth. 2017 Nov 13;17:373. doi: 10.1186/s12884-017-1561-8 (PMC5683371; doi:10.1186/s12884-017-1561-8)
Supplement: Supplementary file 1 — Final baseline questionnaire. Questionnaire used to collect information on pre-frenotomy variables. (PDF 264 kb) [file 12884_2017_1561_MOESM1_ESM.pdf]

Coláiste na Tríonóide, Baile Átha Cliath
Trinity College Dublin
Ollscoil Átha Cliath | The University of Dublin

Frenotomy and associated effect on breastfeeding variables in newborn infants with ankyloglossia (tongue-tie)

BASELINE QUESTIONNAIRE

SEEKING YOUR EXPERIENCES OF BREASTFEEDING

BEFORE

YOUR BABY UNDERWENT FRENOTOMY

Research office use only

Study Number: | | | | |
|---|---|---|---|
| | | | |

Q1. **What is your name?** ____

Q2. **What is your address?** ____

____

____

____

Q3. **What is your mobile telephone number?** ______________________________

Q4. **On what date did your baby have his/her frenotomy procedure?** _____/_____/____

Q5. **How old is your baby now?**

Please write the numbers in the boxes for the whole weeks and any additional days

☐☐	and	☐	
Weeks		Days	

Q6. **Is this your first baby?**

Yes	☐ 1
No	☐ 2

Q7. **Is there a family history of tongue-tie?**

Yes	☐ 1
No	☐ 2

Q8. **In the <u>24-48 hours BEFORE</u> the frenotomy procedure how were you mostly feeding your baby? (*Please tick one option only*)**

Exclusive breastfeeding (breastfeeding only)	☐ 1
Expressing breast milk (feeding using a bottle)	☐ 2
Combination of breast and formula feeding	☐ 3
Combination of breastfeeding and expressed breast milk	☐ 4
Formula feeding only	☐ 5

Q9. Who was the MAIN person that recommended or suggested that your baby should have a frenotomy? ***(please tick one option only)***

I decided myself	☐ 1
Midwife	☐ 2
Public Health Nurse	☐ 3
Private lactation consultant	☐ 4
Doctor/GP	☐ 5
Friend/relative	☐ 6
Member of a local breastfeeding support group	☐ 7
Other *(please describe)*	☐ 8 ____________________

Q10. When your baby latched to the breast in the 24-48 hours BEFORE the frenotomy procedure was he/she able to extend the tongue to the

Lower gum	Yes	☐ 1
	No	☐ 2
Lower lip	Yes	☐ 1
	No	☐ 2

Q11. Please look at the 4 pictures of a baby's tongue and mouth below. Which picture in your opinion best shows what your baby's tongue looked like BEFORE the frenotomoy procedure?

Please indicate by writing the picture number in the box below:

Picture Number: ☐

1.

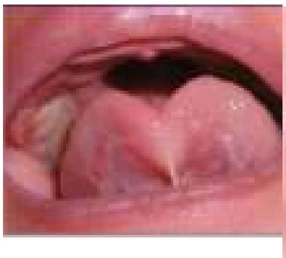

2.

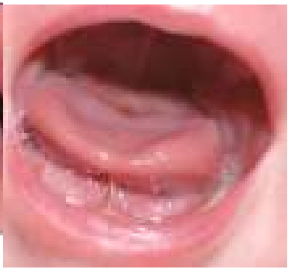

3.

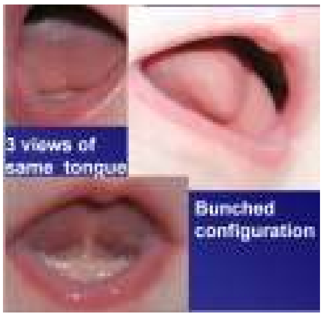

4.

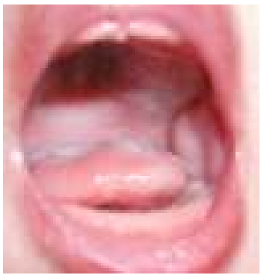

Q12. **<u>Since your baby was born</u> did you experience any of the following**? ***(please tick all that apply)***

Difficulty attaching baby to the breast (how the baby creates a tight seal at the breast)	☐ 1	
Difficulty maintaining attachment	☐ 2	
Breasts feeling full following a feed	☐ 3	
Concern regarding feeding	☐ 4	
Baby unsettled following feeds	☐ 5	
Concern over baby's weight gain	☐ 6	
Concern regarding speech development	☐ 7	
Nipple is mis-shapen following a feed	☐ 8	
Other (please describe)	☐ 9	______________________
None of the above	☐ 10	

Q12a. **If you ticked any of 1-9 in Q12, Did anyone give you help with the difficulty(ies) you were experiencing? *(please tick all that apply*)**

No-one helped me	☐ 1	
Midwife	☐ 2	
Breastfeeding support midwife in hospital helped me	☐ 3	
Public Health Nurse helped me	☐ 4	
Private lactation consultant helped me	☐ 5	
Doctor/GP helped me	☐ 6	
Friend/relative helped me	☐ 7	
Member of local support group	☐ 8	
Other *(please describe)*	☐ 9	______________________

Q13. Which of the following was the <u>MAIN</u> reason you decided your infant should have a frenotomy? *(please tick one option only)*

Mastitis	☐ 1
Recurrent blocked ducts	☐ 2
Cracked nipple	☐ 3
Nipple pain	☐ 4
Nipple misshapen following a feed	☐ 5
Breasts feeling full following a feed	☐ 6
Difficulty at latch on(how the baby takes the breast in his/her mouth)	☐ 7
Baby had difficulty remaining attached to the breast	☐ 8
Baby was unsettled following a feed	☐ 9
Concern regarding later speech development	☐ 10
Other *(please describe)*	☐ 11 ____________________

Q14. Please rate your pain on breastfeeding your infant by circling the number that best describes pain on feeding in the <u>24-48 hours before</u> the frenotomy procedure

<u>0</u> <u>1</u> <u>2</u> <u>3</u> <u>4</u> <u>5</u> <u>6</u> <u>7</u> <u>8</u> <u>9</u> <u>10</u>

No pain Extremely severe
pain

Q15. Latch Scale

For each of the following, please tick <u>ONE</u> box only that <u>BEST</u> describes your infant feeding <u>in the 24-48 hours BEFORE</u> the frenotomy procedure

**Latch**	Too sleepy or reluctant to feed	☐ 1
	Repeated attempts; holds nipple in mouth; needs stimulation to suck	☐ 2
	Grasps breast easily; rhythmic suckling	☐ 3

**Audible swallowing**	None	☐ 1
	A few with stimulation	☐ 2
	Spontaneous and intermittent	☐ 3

**Type of nipple**	Inverted 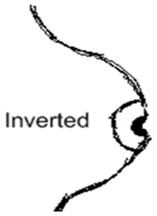	☐ 1
	Flat 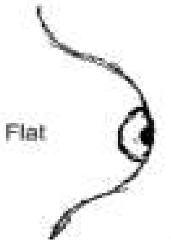	☐ 2
	Everted after stimulation/sucking 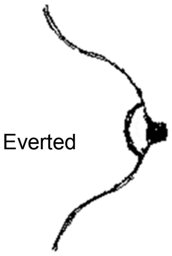	☐ 3
**Nipple shape**	Pinched creases or blanched white after feeding	☐ 1
	Misshapen or change in colour after feeding	☐ 2
	Round, normal colour after feeding	☐ 3
**Breast**	Breasts engorged, rock hard, or large lumps	☐ 1
	Breasts firm, small lumps	☐ 2
	Breasts soft, heavy before feeds	☐ 3
**Nipple**	Nipples cracked, bleeding, large blister(s) and/or bruising	☐ 1
	Nipples reddened, small blister(s) and/or bruising	☐ 2
	Nipples intact	☐ 3
**Urine**	Nappy dry or dark urine	☐ 1
	Nappy damp, urine light yellow	☐ 2
	Nappy wet, urine light yellow or clear	☐ 3

**Satiation**	Crying, fussy, rooting after feeding; after 5 days of age takes longer than 45 to 60 minutes to feed	☐ 1
	Awake, rooting after feeding	☐ 2
	Awake for feeding then relaxes and falls asleep at breast after 10-15 minutes or active feeding with frequent swallows	☐ 3
