# Supplementary material for: Effect of frenotomy on breastfeeding variables in infants with ankyloglossia (tongue-tie): a prospective before and after cohort study
Source: BMC Pregnancy Childbirth. 2017 Nov 13;17:373. doi: 10.1186/s12884-017-1561-8 (PMC5683371; doi:10.1186/s12884-017-1561-8)
Supplement: Supplementary file 2 — Final follow-up questionnaire. Questionnaire used to collect information on post-frenotomy variables. (PDF 239 kb) [file 12884_2017_1561_MOESM2_ESM.pdf]

Coláiste na Tríonóide, Baile Átha Cliath
Trinity College Dublin
Ollscoil Átha Cliath | The University of Dublin

Frenotomy and associated effect on breastfeeding variables in newborn infants with ankyloglossia (tongue-tie)

FOLLOW-UP QUESTIONNAIRE

SEEKING YOUR EXPERIENCES OF BREASTFEEDING

AFTER

YOUR BABY UNDERWENT FRENOTOMY

Research office use only

Study Number: | | | | |

Q1. **What is your name?** ____

Q2. **How old is your baby now?**

Please write the numbers in the boxes for the whole weeks and any additional days

☐☐ and ☐

Weeks Days

Q3. **In the PREVIOUS 24-48 hours describe how you were mostly feeding your baby? (*Please tick one box only*)**

- Exclusive breastfeeding (breastfeeding only) ☐ 1
- Expressing breast milk (feeding using a bottle) ☐ 2
- Combination of breast and formula feeding ☐ 3
- Combination of breastfeeding and expressed breast milk ☐ 4
- Formula feeding ☐ 5

Q4. **If you are no longer breastfeeding (any type), please provide the three main reasons that you decided to stop**

1. ____

2. ____

3. ____

Q5. **When your baby latched to the breast in the PREVIOUS 24-48 hours was he/she able to extend the tongue to the**

Not applicable as no longer breastfeeding ☐ 1

Lower gum
- Yes ☐ 1
- No ☐ 2

Lower lip
- Yes ☐ 1
- No ☐ 2

For questions 6-8, even if you are not breastfeeding NOW; please complete these questions thinking back to when you HAD LAST BEEN breastfeeding your baby after the frenotomy procedure

Q6. In your opinion, do you feel that breastfeeding improved OVERALL since your baby had his/her frenotomy procedure?

Yes ☐ 1
No ☐ 2

If YES, was this

Immediately after the procedure (first few days)
Within two weeks from the procedure ☐ 1
After two weeks from the procedure ☐ 2
☐ 3

Q7. Since your baby had his/her frenotomy procedure did you experience any of the following?
(please tick all that apply)

Difficulty attaching baby to the breast ☐ 1
Difficulty maintaining attachment ☐ 2
Breasts feeling full following a feed ☐ 3
Concern regarding feeding ☐ 4
Baby unsettled following feeds ☐ 5
Concern over baby’s weight gain ☐ 6
Concern regarding speech development ☐ 7
Nipple is mis-shapen following a feed ☐ 8
Other (please describe) ☐ 9 ____________________
None of the above ☐ 10

Q8. If you ticked any of 1-9 in Q7, did anyone give you help with the difficulty(ies) you were experiencing? *(please tick all that apply)*

No-one helped me ☐ 1
Midwife ☐ 2
Breastfeeding support midwife in hospital helped me ☐ 3
Public Health Nurse helped me ☐ 4
Private lactation consultant helped me ☐ 5
Doctor/GP helped me ☐ 6
Friend/relative helped me ☐ 7
Member of local support group ☐ 8
Other *(please describe)* ☐ 9 ____________________

Q9. Please rate your pain on breastfeeding your infant by circling the number that best describes pain on feeding in the <u>24-48 hours AFTER</u> the frenotomy procedure

If you are no longer breastfeeding, please do not circle a number below, and please tick this box ☐

0	1	2	3	4	5	6	7	8	9	10
No pain pain										Extremely severe

Q10. Latch Scale

For each of the following, please tick <u>ONE</u> box only that <u>BEST</u> describes your infant feeding <u>in the 24-48 hours AFTER</u> the frenotomy procedure

If you are no longer breastfeeding, please do not complete below, instead, please tick this box ☐

**Latch**	Too sleepy or reluctant to feed	☐ 1
	Repeated attempts; holds nipple in mouth; needs stimulation to suck	☐ 2
	Grasps breast easily; rhythmic suckling	☐ 3
**Audible swallowing**	None	☐ 1
	A few with stimulation	☐ 2
	Spontaneous and intermittent	☐ 3
**Type of nipple**	Inverted	☐ 1
	Flat	☐ 2
	Everted after stimulation	☐ 3
**Nipple shape**	Pinched creases or blanched white after feeding	☐ 1
	Misshapen or change in colour after feeding	☐ 2
	Round, normal colour after feeding	☐ 3

**Breast**	Breasts engorged, rock hard, or large lumps	☐ 1
	Breasts firm, small lumps	☐ 2
	Breasts soft, heavy before feeds	☐ 3
**Nipple**	Nipples cracked, bleeding, large blister(s) and/or bruising	☐ 1
	Nipples reddened, small blister(s) and/or bruising	☐ 2
	Nipples intact	☐ 3
**Urine**	Nappy dry or dark urine	☐ 1
	Nappy damp, urine light yellow	☐ 2
	Nappy wet, urine light yellow or clear	☐ 3
**Satiation**	Crying, fussy, rooting after feeding; after 5 days of age takes longer than 45 to 60 minutes to feed	☐ 1
	Awake, rooting after feeding	☐ 2
	Awake for feeding then relaxes and falls asleep at breast after 10-15 minutes or active feeding with frequent swallows	☐ 3
